# Tetrahydrobiopterin responsiveness in phenylketonuria: prediction with the 48-hour loading test and genotype

**DOI:** 10.1186/1750-1172-8-103

**Published:** 2013-07-10

**Authors:** Karen Anjema, Margreet van Rijn, Floris C Hofstede, Annet M Bosch, Carla EM Hollak, Estela Rubio-Gozalbo, Maaike C de Vries, Mirian CH Janssen, Carolien CA Boelen, Johannes GM Burgerhof, Nenad Blau, M Rebecca Heiner-Fokkema, Francjan J van Spronsen

**Affiliations:** 1 Division of Metabolic Diseases, University Medical Center Groningen, Beatrix Children’s Hospital CA33, PO box 30.001, Groningen 9700 RB, The Netherlands; 2 University Medical Center Utrecht, Wilhelmina Children’s Hospital, Utrecht, The Netherlands; 3 Academic Medical Center, University Hospital of Amsterdam, Amsterdam, The Netherlands; 4 Maastricht University Medical Center, Maastricht, The Netherlands; 5 Radboud University Nijmegen Medical Center, Nijmegen, The Netherlands; 6 Leiden University Medical Center, Leiden, The Netherlands; 7 University Children’s Hospital, Heidelberg, Germany; 8 University Children’s Hospital, Zürich, Switzerland

**Keywords:** Phenylketonuria, PKU, Tetrahydrobiopterin, Sapropterin dihydrochloride, Pharmacological chaperone, Genotype, Loading test

## Abstract

**Background:**

How to efficiently diagnose tetrahydrobiopterin (BH4) responsiveness in patients with phenylketonuria remains unclear. This study investigated the positive predictive value (PPV) of the 48-hour BH4 loading test and the additional value of genotype.

**Methods:**

Data of the 48-hour BH4 loading test (20 mg BH4/kg/day) were collected at six Dutch university hospitals. Patients with ≥30% phenylalanine reduction at ≥1 time points during the 48 hours (potential responders) were invited for the BH4 extension phase, designed to establish true-positive BH4 responsiveness. This is defined as long-term ≥30% reduction in mean phenylalanine concentration and/or ≥4 g/day and/or ≥50% increase of natural protein intake. Genotype was collected if available.

**Results:**

177/183 patients successfully completed the 48-hour BH4 loading test. 80/177 were potential responders and 67/80 completed the BH4 extension phase. In 58/67 true-positive BH4 responsiveness was confirmed (PPV 87%). The genotype was available for 120/177 patients. 41/44 patients with ≥1 mutation associated with long-term BH4 responsiveness showed potential BH4 responsiveness in the 48-hour test and 34/41 completed the BH4 extension phase. In 33/34 true-positive BH4 responsiveness was confirmed. 4/40 patients with two known putative null mutations were potential responders; 2/4 performed the BH4 extension phase but showed no true-positive BH4 responsiveness.

**Conclusions:**

The 48-hour BH4 loading test in combination with a classified genotype is a good parameter in predicting true-positive BH4 responsiveness. We propose assessing genotype first, particularly in the neonatal period. Patients with two known putative null mutations can be excluded from BH4 testing.

## Introduction

In phenylketonuria (PKU, OMIM 261600), deficiency of the enzyme phenylalanine hydroxylase (PAH) leads to increased phenylalanine (Phe) concentrations. Untreated, this results in progressive and irreversible cerebral damage [1,2]. For almost 60 years, a life-long low-Phe diet has been the only possible treatment for PKU. Dietary treatment has proved to be very effective in preventing the devastating consequences of PAH deficiency when started early in life (e.g. after neonatal screening). The cognitive outcome strongly depends on optimal metabolic control [3,4]. Unfortunately, adherence to such a diet proves difficult [5].

A pharmaceutical formulation of tetrahydrobiopterin (BH4), the natural co-factor and co-substrate of PAH, is now an FDA and EMA-registered drug (sapropterin dihydrochloride; Kuvan®) and provides a new treatment option in a significant number of patients. It acts as a pharmacological chaperone by stabilizing PAH [6]. In BH4-responsive patients, BH4 decreases the blood Phe concentration and/or increases the dietary Phe tolerance [7,8]. Correct and efficient identification of BH4-responsive patients is important, both to improve the fast assessment, as well as to avoid false expectations and unnecessary costs. Unfortunately, there is still no golden standard on how to assess BH4 responsiveness most efficiently.

Three methods have been proposed for the prediction of BH4 responsiveness: the 7–28 days BH4 challenge [9,10], the 48-hour BH4 loading test [11] and, the START (sapropterin therapy actual response test) BH4 challenge and genotyping protocol [12]. Genotype was frequently reported to be useful in predicting or excluding BH4 responsiveness [8,13-15]. However, only small studies have correlated genotype data with *in vivo* BH4 challenge tests and long-term BH4 responsiveness [16-29].

To improve the assessment of BH4 responsiveness, we investigated the positive predictive value (PPV) of the 48-hour BH4 loading test and additional value of genotype for BH4 responsiveness in a large cohort of Dutch PAH-deficient patients.

## Methods

### Subjects and protocol

In a national collaborative study, data were collected from 183 patients who participated in the 48-hour BH4 loading test between November 2009 and December 2010 in six Dutch university medical centres. Following the European Medicines Agency (EMA) and the Dutch regulations on BH4 prescriptions, only patients above four years of age, requiring a Phe restricted diet, and not pregnant or planning a pregnancy were considered for treatment with BH4. The 48-hour BH4 loading test was also performed in younger children. The protocol consisted of two parts: the 48-hour BH4 loading test to assess ‘potential BH4 responsiveness’ and the BH4 extension phase to establish ‘true-positive BH4 responsiveness’. The BH4 protocol (the 48-hour BH4 loading test and the BH4 extension phase) was considered standard patient care by the Medical Ethical Committee of the University Medical Center Groningen.

The 48-hour BH4 loading test was largely based on recommendations made by the European working group on PKU [11], requiring baseline Phe concentrations over 400 μmol/L. Dutch treatment guidelines recommend blood Phe concentrations of 120–360 μmol/L in patients under twelve years of age and 120–600 μmol/L in patients over twelve years of age [30]. Therefore, well-controlled patients with Phe concentrations within the recommended range had to be supplemented with dietary Phe (L-Phe in powder, a protein rich supplement based on milk protein, or an increase in natural protein intake) to reach a stable Phe concentration ‘just’ over 400 μmol/L. The extra Phe intake was used until the test was finished. Typically, multiple blood samples were taken to assure stable Phe concentrations. Patients with Phe concentrations above 400 μmol/L were instructed to continue their usual diet. All patients received 20 mg/kg BH4 (sapropterin dihydrochloride; Kuvan®) twice, directly after blood sampling at baseline (T = 0) and after 24 hours (T = 24). BH4 dosages were rounded up or down to the nearest 100 mg. Blood samples were collected after T = 0, 8, 16, 24 and 48 hours. Patients with a 30% or more reduction in blood Phe concentration at one or more points compared to the baseline (T = 0) were regarded as ‘potential BH4-responsive’ and were invited for the BH4 extension phase.

The BH4 extension phase consisted of three steps in which the blood Phe concentrations, which were measured one to three times a week, had to remain within the ranges of the guideline. Beforehand, a three-day dietary record was used to determine baseline Phe tolerance. BH4 was then introduced at 20 mg/kg/day, followed by: an increase of dietary Phe (to reach the maximal Phe tolerance), BH4 dose adjustment (decrease if possible) and finally adjustment to the Phe-free amino acid supplement according to the patients’ total protein needs (age and sex dependent). Following this last step, if blood Phe concentrations exceeded the upper target limit, both the BH4 dose and the amount of Phe intake were re-evaluated. Different methods were used to increase dietary Phe (natural protein or L-Phe powder/protein rich supplement first), but in all cases this was done gradually and on an individual basis. The method to determine the maximum Phe tolerance was not protocolized, although it was advised to keep or bring the Phe concentrations within treatment range. Typically, when Phe levels were above the target concentration, natural protein intake was decreased by 10-20% depending on the amount of increase in Phe concentration and the absolute concentration. The reduction of the Phe-free amino acid supplement was performed in multiple steps in case of large quantities. When the patients had Phe concentrations within target range or were stabile, a three-day dietary record was used to determine the final Phe tolerance and dietary sufficiency for all nutrients. Dietary records were analysed by nutritionists qualified in metabolic diseases at the various centres. True-positive BH4 responsiveness was defined as a 30% or more reduction in blood Phe concentration compared to mean blood Phe concentrations prior to the 48-hour BH4 loading test with the same diet, and/or an increase in dietary Phe tolerance of ≥50% or ≥4 grams of natural protein without increasing the Phe concentrations above the upper target.

### Assessments

Blood Phe concentrations in dried blood spots were measured by the patients’ respective centre laboratory according to standard quantitative methods, with the same method per patient. To calculate the individual mean blood Phe concentration prior to the BH4 introduction, all known values from the year preceding the BH4 loading test were used, with a minimum of four measurements; alternatively, the period was extended to two years or this value was considered missing. The individual mean blood Phe concentration after the BH4 extension phase was calculated from all the Phe concentrations in the first three months after finishing the BH4 extension phase, with a minimum of four measurements. The period was extended until four samples were available in patients that did not send that many samples (maximum one year). Weight was usually measured at the first day of the BH4 loading test or at a recent outpatient visit, to determine the total daily dose of BH4. Otherwise, the most recent value was used. The medical history was obtained to ascertain the blood Phe concentration at diagnosis. Data on genotype were collected if available. A Medline database literature search was performed to compose a list of mutations associated with long-term BH4 responsiveness. This list consisted of thirty mutations. Mutations with a known residual *in vitro* activity of ≤1%, all nonsense mutations, variants altering the reading frame and splice-site mutations leading to exon skipping and disruption of the reading frame were regarded putative null mutations [13,14,20]. All genotypes are tabulated in the BIOPKU database (http://www.biopku.org) and compared with existing information.

### Statistical analysis

Normality was defined according to the Shapiro Wilk test. As almost all data showed skewed distributions, data are presented as medians with inter-quartile ranges (IQR). The Mann–Whitney U test was used to compare independent continuous data. To compare related continuous data the Wilcoxon Signed Rank test was used. The Chi-square test was used for categorical data unless the minimum expected count was smaller than five, in which case Fisher’s exact test was used. Statistical analyses were performed using PASW statistics version 18.0.3, SPSS, Inc., Chicago, IL, USA. A two-tailed P-value < 0.05 was assumed as statistically significant.

## Results

### Cohort

**Figure 1:**
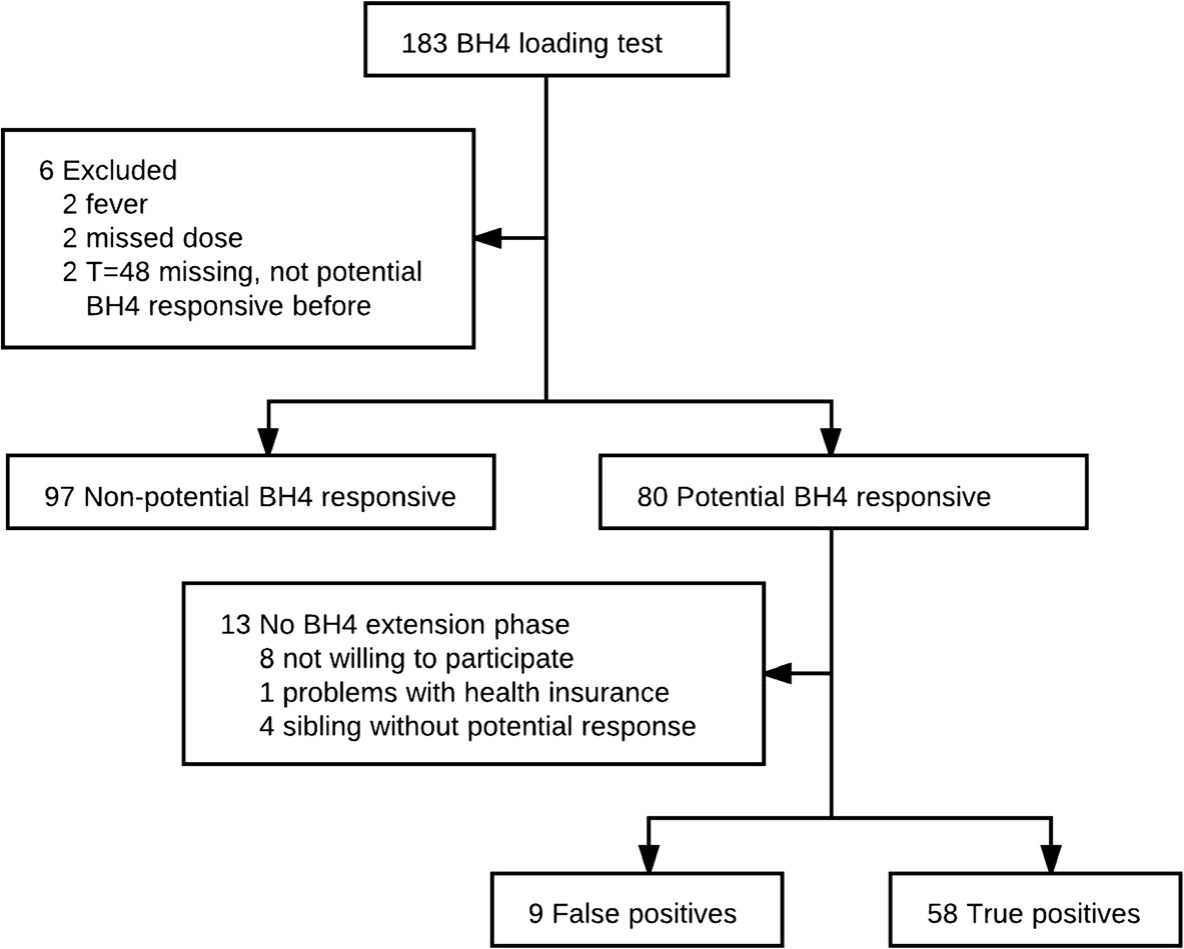
Study profile.

A total of 183 patients started the 48-hour BH4 loading test. Six patients were excluded because of illness, missing T = 48 blood sample or irregular BH4 administration (Figure 1). The median (IQR) age of the 177 patients that successfully completed the 48-hour loading test was 13.8 (8.6-21.1) years. Seven children were younger than four years old during the 48-hour BH4 loading test, but none during the BH4 extension phase. A female gender accounted for 52.5% of the study population. Phe was supplemented in 51 out of 66 patients younger than 12 years old and in 36 out of 111 patients of 12 years or older.

### 48-hour BH4 loading test and BH4 extension phase

In the 48-hour BH4 loading test, 80 out of 177 (45.2%) patients were potentially BH4-responsive. Out of 80 patients with potential BH4 responsiveness, 67 (85%) started the BH4 extension phase. False-positive BH4 responsiveness was shown in nine of them. Therefore, the PPV of the 48-hour BH4 loading test was 87%. Demographic and clinical details of all patients with no potential BH4 responsiveness, false-positive and true-positive BH4 responsiveness as well as potentially responsive patients who did not participate in the BH4 extension phase are shown in Table 1.

**Table 1:** Demographic and clinical details of patients with non-potential BH4 responsiveness and False/True-positives

**BH4 loading test:**	**Non-potential BH4-responsive (n = 97)**	**Potential responder (n = 80)**		
**BH4 extension phase:**		**False-positive (n = 9)**	**True-positive (n = 58)**	**Other**^**1 **^**(n = 13)**
Sex				
Male	47 (48.5)	6 (66.7)	24 (41.4)	7 (53.8)
Female	50 (51.5)	3 (33.3)	34 (58.6)	6 (46.2)
Age (years) A*	14.7 (10.3 – 23.0)	8.0 (6.4 – 11.9)	12.3 (7.7 – 17.9)	14.1 (10.4 – 19.4)
<12 years A**	30 (30.9)	7 (77.8)	25 (43.1)	4 (30.8)
≥12 years	67 (69.1)	2 (22.2)	33 (56.9)	9 (69.2)
Baseline Phe (μmol/L)^2^B*** C*	663 (530 – 915)	620 (528 – 831)	465 (358 – 555)	485 (386 – 590)
Mean Phe^3,4^B*** C**	494 (364 – 700)	455 (406 – 553)	304 (244 – 401)	267 (239 – 407)
<12 years B* C**	397 (290 – 441)	455 (372 – 539)	282 (227 – 400)	274 (207 – 318)
≥12 years^4^B***	564 (427 – 773)	440, 759 (n = 2)	332 (255 – 414)	264 (239 – 456)
Phe at diagnosis^5^B***, C***				
<600 μmol/L	3 (3.5)	0 (0.0)	26 (44.8)	4 (30.8)
600–1199 μmol/L	13 (15.3)	2 (22.2)	26 (44.8)	3 (23.1)
>1200 μmol/L	69 (81.2)	7 (77.8)	6 (10.3)	6 (46.2)

Data are n (%) or median (interquartile range). ^1^ no BH4 extension phase. ^2^ Phe concentration at T = 0 (with Phe loading when necessary). ^3^ Mean Phe concentration prior to the BH4 loading test. ^4^ Missing in n = 14 (<30%), n = 10 (True-positives) and n = 1 (Other). ^5^ First Phe concentration in hospital, missing in n = 12 (<30%). A) comparing non-potential BH4-responsive and false-positive, B) comparing non-potential BH4-responsive and true-positive, C) comparing false-positive and true-positive. *P = <0.05, **P = <0.01, ***P = <0.001.

In patients with a 30-40% decrease in Phe concentrations (n = 18), the PPV was 67%, while this was 94% in patients with ≥40% reduction (n = 49), P = 0.009. The PPV of patients responding only at T = 48 (n = 11) was lower than patients who respond within 24 hours (n = 56) (PPV 64% and 91% respectively, P = 0.03). Patients who responded within 24 hours showed a median (IQR) Phe concentration decrease of 56 (42 – 57) percent at the 24 hour time point, whereas this was 24 (16 – 26) percent in patient who responded only at T = 48. At T = 48 this was 50 (35 – 68) and 36 (34 – 45) percent, respectively. Patients with at least 30% decrease in Phe concentration at two or more moments showed true BH4 responsiveness in 47 out of 50, whilst this was ten out of 16 in patients with only one moment. Five patients had this one moment at another time point than T = 48, of them 3 showed true BH4 responsiveness.

**Figure 2:**
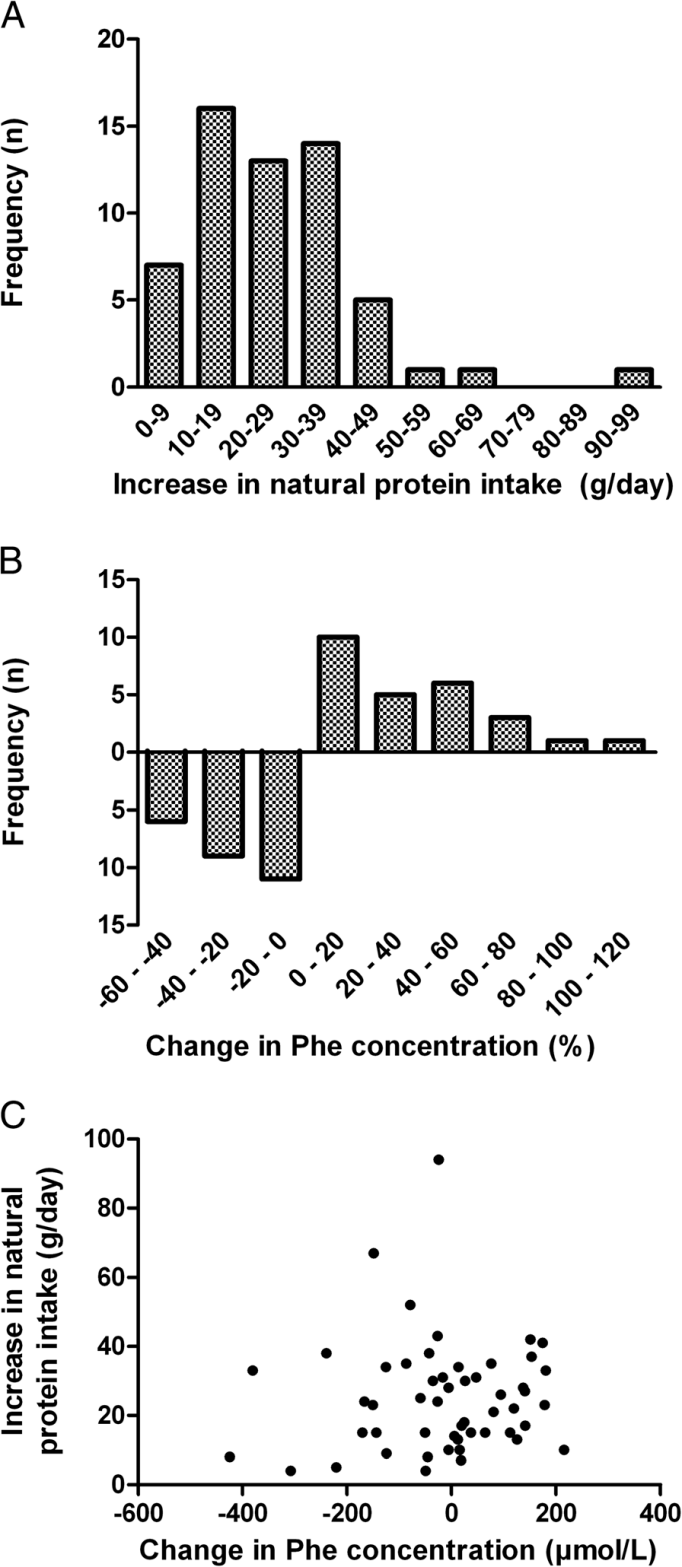
**Treatment results in patients with true-positive BH4 responsiveness. (A)** Histogram of the increase in natural protein intake (g/day). **(B)** Histogram of the percentage change in phenylalanine concentration. **(C)** Combined overview of the increase in natural protein intake (g/day) and the absolute change in phenylalanine concentration.

The duration of the BH4 extension phase ranged from six to 76 weeks in patients with true-positive BH4 responsiveness with a median of 24 weeks. During this period a median (IQR) amount of 40 (24 – 55) blood samples were taken per patient. For patients with a false-positive response the duration of the BH4 extension phase was 6 to 43 weeks, with a median of 13 weeks. In patients with true-positive BH4 responsiveness the median (IQR) natural protein intake increased significantly from 17 (12–28) g to 49 (34 – 57) g (P = 0.000). The distribution of increase in natural protein is shown in Figure 2A. In 41 patients four or more blood samples were taken within three months after stabilization (with a median of seven samples). Eleven patients collected four blood samples within one year, with a mean period of 20 ± 6.2 weeks. In six (adult) patients too little samples were collected to calculate the mean Phe concentration after stabilization of BH4 treatment. These six patients were excluded from Phe concentration analysis. The median (IQR) of the mean Phe concentrations in patients with enough samples did not significantly change (350 (250–460) μmol/L prior to BH4 treatment and 329 (272–415) μmol/L after stabilization, P = 0.716). The distribution of the decrease in Phe concentration is shown in Figure 2B. An overview of changes in natural protein as well as Phe concentration can be seen in Figure 2C. Patients with a late response during the 48-hour BH4 loading test and who were true BH4 responders showed no difference in baseline natural protein intake compared to patients that responded within 24 hours (Median (IQR) 13 (7 – 21) g versus 17 (12 – 29) g, respectively, P = 0.211). Also the median amount of increase was not different (24 g and 25 g of natural protein respectively, P = 0.674).

### Genotype

Genotype was determined in 127 patients and revealed heterozygosity in 102 patients and homozygosity in 18 patients, while only one mutation was identified in four patients and no PAH mutations were identified in two patients (siblings). Furthermore, one patient was found to have a silent mutation (p.V245V) and a p.G272* mutation, with no evidence of a third mutation. This patient had a severe phenotype (diagnostic Phe concentration of 1963 μmol/L and a dietary Phe tolerance of 8.1 mg/kg/day) and was potentially BH4-responsive (47% Phe reduction), but did not want to participate in the BH4 extension phase. An overview of all 120 complete genotypes including 48-hour BH4 loading test and BH4 extension phase results is available in the Additional file 1: Table S1. The table also shows four novel mutations of the PAH gene. An overview of the mutations associated with long-term BH4 responsiveness is shown in Table 2.

**Table 2:** Mutations associated with true BH4 responsiveness and mutations newly associated with true BH4 responsiveness

p.F39L	p.V190A	p.K320N	**New**
p.L48S	p.V230I	p.L348V	p.A104D
p.F55L	p.R241Q	p.P366H	p.T193I
p.I65T	p.R241H	p.A373T	p.P211T
p.R68S	p.R241C	p.E390G	p.T238A
p. D129G	p.V245A	p.A403V	p.P314H
p.P147L	p..P275S	p.F410S	p.R408Q
p.V177L	p.A300S	p.Y414C	
p.V177M	p.A309V	p.D415N	
p.E178G	p.A313T	p.P416Q	

**Figure 3:**
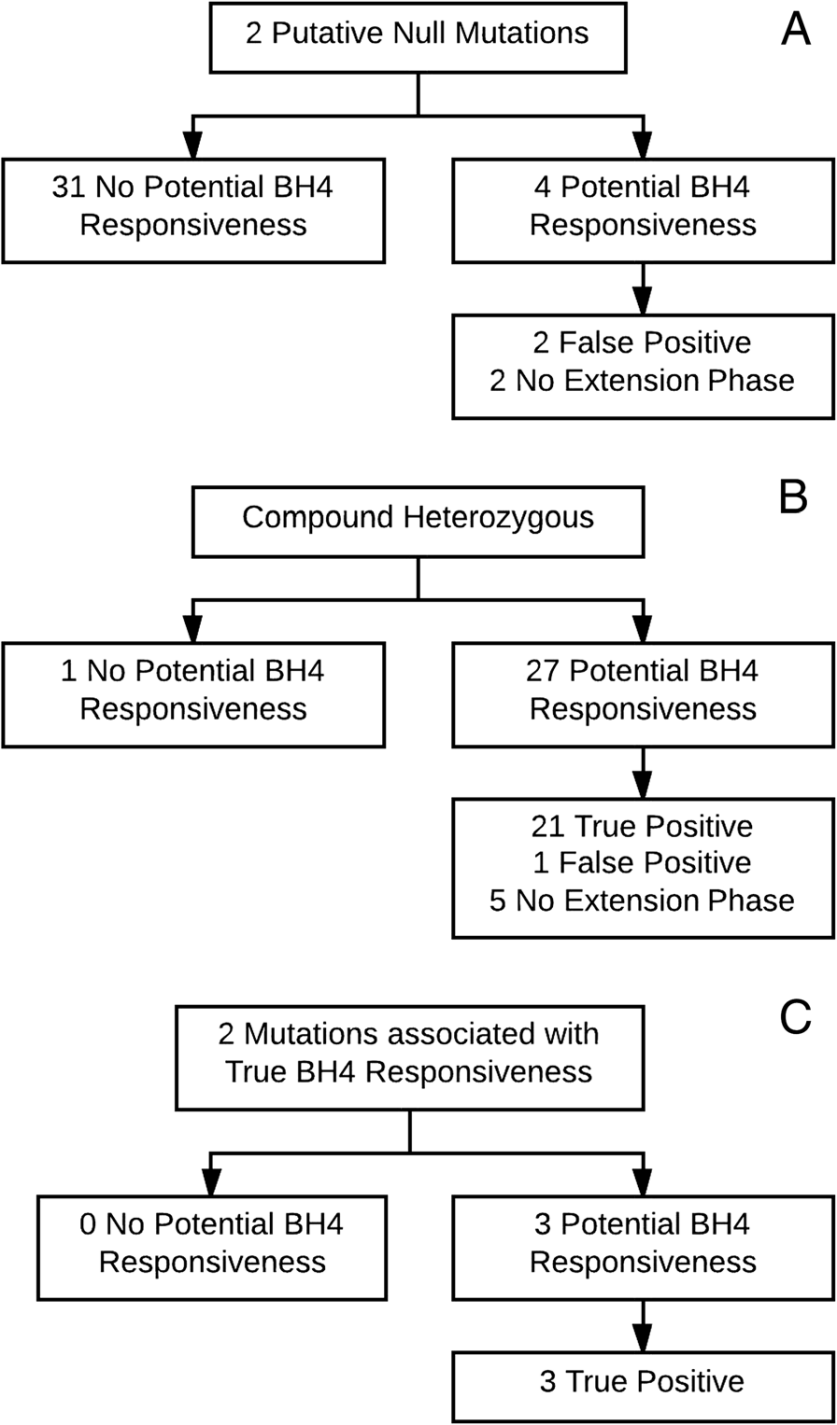
**Outcome.** The outcome of the 48-hour BH4 loading test and the BH4 extension phase in patients with **(A)** 2 Putative Null mutations **(B)** Compound heterozygosity **(C)** 2 mutations associated with true BH4 responsiveness.

Thirty-five patients had a genotype consisting of two putative null mutations, 28 a functionally hemizygous genotype (a combination of a putative null mutation on one allele and a mutation associated with true BH4 responsiveness on the other allele) and, three patients a combination of two mutations associated with true BH4 responsiveness. For these patients the outcome is shown in Figure 3.

Seven patients with true-positive BH4 responsiveness were identified as having a genotype consisting of a putative null mutation and a mutation not yet associated with BH4 responsiveness or a homozygous genotype with mutations not yet associated with BH4 responsiveness (p.A104D, p.T193I, p.P211T, p.P314H, p.R408Q and the novel mutation p.T238A). One or more unclassified mutations including the p.R261Q and p.R158Q mutation were detected in 47 patients. Results regarding the 48-hour BH4 loading test and the BH4 extension phase are shown in Table 3.

**Table 3:** Genotypes with unclassified mutations

**No potential BH4 responsiveness (n)**	**Potential BH4 responsiveness (n)**
c.168+5G>C^1^/p.R261Q (1)	**False positive**
c.842+1G>A^1^/p.R243Q (1)	c.1315 + 1G > A^1^/p.I174N (1)
c.842+5G>A^1^/p.R158Q (1)	p.R158Q/p.R158Q (1)
c.1066-11G>A^1^/p.D222G (1)	p.R158Q/p.Y277D (1)
c.1315+1G>A^1^/p.I174N (1)	p.R243*^1^/p.R261Q (1)
c.1315+1G>A^1^/p.R261Q (4)	
c.1315+1G>A^1^/p.R261P (1)	**True positive**
c.1315+1G>A^1^/p.Y277D (1)	p.L48S^2^/p.R408W (1)
c.1315+1G>A^1^/p.R408W (1)	p.A104D^3^/p.R158Q (1)
p.L48S^2^ /p.R261Q (1)	p.A104D^3^/p.R408W (1)
p.R111X^1^/p.R408W (1)	p.R158Q/p.V190A^2^ (2)
p.R158Q/p.R158Q (1)	p.R158Q/p.Y414C^2^ (2)
p.R158Q/p.R243*^1^ (1)	p.I174N/p.P211T^3^ (1)
p.R158Q/p.R261P (1)	p.E178G^2^/p.Y343C (1)
p.R158Q/p.R408W^1^ (1)	p.R261Q/p.P281L^1,4^ (1)
p.I174T/p.F299C^1^ (1)	p.R261P/p.Y414C^2^ (1)
p.C203Y/ p.L348V^2^ (1)	p.Y277D/p.P314H^3^ (1)
p.R261Q/p.R261Q (2)	p.E280K/p.Y414C^2^ (1)
p.R261Q/p.E280K^1^(2)	p.A300S^2^/p.R408W (1)
p.R261Q/p.P281L^1^ (1)	
p.P281L^1^/p.R413P (2)	**No extension phase**
	p.L48S^2^/p.R158Q (1)
	p.Y277D/p.P314H^3^ (1)

^1^ Putative Null Mutation. ^2^ Mutation associated with true BH4 responsiveness.

^3^ New Responsive Mutation. ^4^ Natural protein increase: 6.5 g/day (before treatment 10.5 g/day) with no statistically significant Phe concentration increase [6%, P = 0.379].

Of the 44 patients with at least one mutation known to be associated with BH4 responsiveness, 41 showed potential BH4 responsiveness (three non-potential responders: c.1066-11G>A/p.L48S 27%, p.L48S/p.R261Q 28%, p.C203Y/p.L348V 25%). Of 41 potential BH4 responders 34 patients participated in the BH4 extension phase. Of those 34 patients with potential responsiveness and a favourable genotype, 33 were true responsive (97%, no true BH4 responsiveness was seen in p.I65T/p.L311P).

## Discussion

This study, in a large cohort of PKU patients, shows that the 48-hour BH4 loading test has a high PPV for true BH4 responsiveness. Furthermore, it shows that patients with genotypes including at least one mutation known to be associated with long-term BH4 responsiveness have a high rate of true response. This is the first study to investigate the PPV for BH4 responsiveness of the 48-hour BH4 loading test and the additional value of genotype. Establishing a reliable pre-selection method is of high importance, since it could save time and money, and will prevent false expectations. The treatment of BH4 responsive PKU patients has been markedly changed since the therapy was introduced. Expansion of the Phe-restricted diet or even complete liberation can reduce a major burden. Although quality of life results obtained by standardized questionnaires (not suitable for PKU) do not show a significant improvement, patients do report an increased quality of life [27,28].

Recently, we amongst others suggested that 24 hours is not long enough to detect patients with a delayed response to BH4 [16,31-33]. Indeed in this study we confirm that patients with true BH4 responsiveness can be missed in a 24 hour test. Other studies suggest extending the BH4 loading test to beyond 48 hours [9,28,34]. We detected some patients who tend to show a response at T = 48. However, we did not compare our results to a longer lasting test, such as the 7–28 day BH4 challenge. As our data indicate that patients who show a first response at T = 48 are less likely to show true BH4 responsiveness than patients who respond within 24 hours, prolongation of the BH4 loading test will possibly increase the number of patients with true BH4 responsiveness, but will also decrease the PPV and specificity. Also, influence of external factors increases with the length of the test. Moreover, patients with only one moment of response during the 48-hour BH4 loading test can show true BH4 responsiveness. Another strategy is published by Singh and Quirk. They chose to see whether it is possible to liberalize the diet in patients with just 15% decrease of Phe after one month of BH4 treatment [35]. Unfortunately however, this study does not show whether such low a cut off results in the detection of extra BH4 responsive patients.

As expected blood Phe concentrations at the diagnosis, baseline Phe concentrations and Phe concentrations prior to the BH4 loading test were significantly lower in patients with true-positive BH4 responsiveness than patients without potential BH4 responsiveness and false-positives. Nevertheless, these biochemical parameters can give an indication but show too much overlap for practical use in predicting BH4 responsiveness. Another notable finding was that a majority of false-positive patients are found in the younger age range. At present, we do not have a clear explanation for this finding. Possibly, younger patients have more variability in their blood Phe concentrations.

Since a few years it is recognized that a genotype can be helpful in predicting BH4 responsiveness and to do so a full genotype is required [14,15,36]. Our results underline this assumption. Patients who have a functionally hemizygous genotype are frequently true BH4 responders. The three patients who were not potential BH4 responders all reached a 20-30% Phe concentration decrease in the 48-hour BH4 loading test and can potentially also benefit from the therapy. The patients with two mutations associated with true BH4 responsiveness were all true BH4 responders. This concerns only three patients as patients with this genotype combination probably have mild hyperphenylalaninemia very often and do not always require treatment, which was an inclusion criterion. Furthermore, the results in this study support the suggestion in the literature that patients with two putative null mutations are not candidates for BH4 treatment [36,37]. In a recent study by Sterl et al. the proportions of predicted BH4 responsiveness are fairly similar to our results [38].

An important remark is that a classified genotype is needed to predict BH4 responsiveness. As more than 400 missense mutations are described and 75 to 90 percent of the patients are compound heterozygous [8,13,27], this results in a large number of as yet unclassified genotypes. Additionally, some mutations are associated with an inconsistent BH4 response, for instance p.R158Q [8,13], p.I65T [21,26,39,40] and p.R261Q [8,13,14,27]. In our patients, where the p.R158Q mutation was homozygous or combined with a putative null mutation, it showed no BH4 responsiveness. The p.I65T mutation combined with a putative null mutation showed potential response in two out of two patients. However, unfortunately, one patient did not perform the BH4 extension phase (60% Phe concentration decrease) and the other was a false-positive. The p.R261Q mutation again showed variable results, with a response just under the threshold of 30% remarkably often. Patients who are functionally hemizygous for p.R261Q seldom show true BH4 responsiveness, but one of our patients and one patient described by Hennermann et al. [27], both p.R261Q/L.P281L, show (modest) long-term BH4 responsiveness.

Since this study reports patient care results, patients without potential BH4 responsiveness did not participate in the BH4 extension phase. This limits the conclusion regarding its diagnostic value. Other results, however (number of false-positives in patients with borderline response), support the presumption that the number of false-negatives will be limited. A possible bias in the 48-hour BH4 loading test could be the supplementation of Phe to increase the baseline Phe concentration and the different methods to reach this increase. The influence of the different metabolic kinetics is unclear [41,42]. With respect to the true BH4 responsiveness criteria, the extra 4 g of natural protein (180 mg Phe) tolerance increase criterion can be considered relatively modest. This age independent criterion was agreed within the Dutch group of metabolic physicians. If the criterion were doubled to 8 g of natural protein, four patients would not have met this tolerance requirement. Two out of these four patients also showed a 30% or more decrease in blood Phe concentration. Of the other two patients (both children), genotype showed heterozygosity for p.R261Q/p.P281L in one and c.1315+1G>A/p.L348V in the other. Furthermore, we anticipated that the BH4 extension phase would take less than six months. However, the actual duration exceeded this six months time period in 41 percent of the patients, with three patients taking even longer than one year. There are several explanations for this longer duration of which the exploration of the possible speed of Phe tolerance increase is an important one, as is the motivation of the patients during the last stages (especially blood sampling frequency).

## Conclusions

In conclusion, notwithstanding the small number of patients, a classified genotype seems a good predictor of BH4 responsiveness. Therefore, assessing genotype first maybe an option, particularly in the neonatal period when in Europe BH4 is not yet approved for use. In case the protocol would be altered to assessing genotype first, the 48-hour BH4 loading test is an adequate and patient-friendly method for detecting BH4 responsiveness, especially for patients with an unclassified or unknown genotype. The duration of the test is adequate for many patients and whereas multiple time points with ≥30% Phe concentration reduction are beneficial, one time point can indicate true BH4 responsiveness.

## Abbreviations

BH4: Tetrahydrobiopterin; IQR: Interquartile range; PAH: Phenylalanine hydroxylase; Phe: Phenylalanine; PKU: Phenylketonuria; PPV: Positive predictive value.

## Competing interests

KA was financially supported by a MD/PhD grant from the Junior Scientific Master Class of the University of Groningen and received research funding from Merck Serono. MvR has received research grants, consultancy fees and advisory-board fees from Danone Research and Merck Serono, speaker’s honoraria from Danone Research, Merck Serono and Orphan Europe, and expert testimony fees from Merck Serono. FCH has received travel support from Merck Serono. AMB has received research grants from Danone Research, speaker’s honoraria and advisory-board fees from Merck Serono and Danone Research. NB has received advisory-board fees, consultancy fees and research grants from Merck Serono and Biomarin. FJvS has received research grants, advisory-board fees and speaker’s honoraria from Merck Serono and Nutricia Research.

## Authors’ contributions

FJvS and MvR contributed to protocol design. KA, MvR, FCH, AMB, CEMH, MERG, MCdV, MCHJ, CCAB and FJvS were responsible for the clinical follow-up and provided research data. KA contributed to the data processing and statistical analysis. All authors interpreted the data, specifically JGMB the statistical data, NB the genetic data and MRHF the biochemical data. KA drafted the paper. All authors read and approved the final manuscript.

## Supplementary Material

Additional file 1: Table S1 Overview of genotypes, results of the 48-hour BH4 loading test and the BH4 extension phase. Click here for file
