# Supplementary material for: Tetrahydrobiopterin responsiveness in phenylketonuria: prediction with the 48-hour loading test and genotype
Source: Orphanet J Rare Dis. 2013 Jul 10;8:103. doi: 10.1186/1750-1172-8-103 (PMC3711849; doi:10.1186/1750-1172-8-103)
Supplement: Additional file 1: Table S1 — Overview of genotypes, results of the 48-hour BH4 loading test and the BH4 extension phase. [file 1750-1172-8-103-S1.pdf]

Supplementary Table: Overview of genotypes, results of the 48-hour BH4 loading test and the BH4 extension phase

Allele 1		Allele 2		Phe load†	T=0	Max Phe ↓ (%)	Extension phase	
IVS2+5G>C‡§	c.168+5G>C	p.W187*‡	c.561G>A	No	741	4	No	
IVS2+5G>C‡§	c.168+5G>C	p.W187*‡	c.561G>A	No	326	17	No	
IVS2+5G>C‡	c.168+5G>C	p.R243*‡	c.727C>T	No	1049	25	No	
IVS2+5G>C‡	c.168+5G>C	p.R261Q¶	c.782G>A	No	491	15	No	
**IVS5-21del177‡**	c.510-21_665del	p.G272*‡	c.814G>T	No	855	5	No	
IVS7+1G>A‡	c.842+1G>A	p.R243Q‡	c.728G>A	Yes	626	5	No	
IVS7+5G>A‡	c.842+5G>A	p.R158Q¶	c.473G>A	Yes	896	-1	No	
IVS7+5G>A‡	c.842+5G>A	p.Y414C||	c.1241A>G	No	503	36	Yes	positive
**IVS7-5T>C‡**	c.843-5T>C	p.P281L‡	c.842C>T	No	949	22	No	
IVS10-11G>A‡	c.1066-11G>A	IVS10-11G>A‡	c.1066-11G>A	No	1224	22	No	
IVS10-11G>A‡	c.1066-11G>A	IVS10-11G>A‡	c.1066-11G>A	Yes	550	14	No	
IVS10-11G>A‡	c.1066-11G>A	IVS10-11G>A‡	c.1066-11G>A	No	705	3	No	
IVS10-11G>A‡§	c.1066-11G>A	p.L48S||	c.143T>C	Yes	478	56	No	
IVS10-11G>A‡§	c.1066-11G>A	p.L48S||	c.143T>C	No	845	27	No	
IVS10-11G>A‡§	c.1066-11G>A	p.L48S||	c.143T>C	Yes	459	48	No	
IVS10-11G>A‡	c.1066-11G>A	p.D222G¶	c.665A>G	Yes	936	23	No	
IVS12+1G>A‡	c.1315+1G>A	IVS12+1G>A‡	c.1315+1G>A	No	769	9	No	
IVS12+1G>A‡	c.1315+1G>A	p.S16* ‡	c.47_48delCT	Yes	715	-4	No	
IVS12+1G>A‡	c.1315+1G>A	p.S16* ‡	c.47_48delCT	No	451	12	No	
IVS12+1G>A‡	c.1315+1G>A	p.S16* ‡	c.47_48delCT	No	599	0	No	
IVS12+1G>A‡	c.1315+1G>A	p.L48S||	c.143T>C	Yes	304	69	Yes	positive
IVS12+1G>A‡	c.1315+1G>A	p.I65T||	c.194T>C	Yes	587	60	No	
IVS12+1G>A‡§	c.1315+1G>A	p.I174N¶	c.521T>A	Yes	692	35	Yes	negative
IVS12+1G>A‡§	c.1315+1G>A	p.I174N¶	c.521T>A	Yes	663	17	No	
IVS12+1G>A‡	c.1315+1G>A	p.W187*‡	c.561G>A	No	514	7	No	
IVS12+1G>A‡§	c.1315+1G>A	p.P211T‡‡	c.631C>A	Yes	367	81	Yes	positive
IVS12+1G>A‡	c.1315+1G>A	p.R241C||	c.721C>T	Yes	464	45	Yes	positive
IVS12+1G>A‡	c.1315+1G>A	p.R243*‡	c.727C>T	Yes	588	21	No	
IVS12+1G>A‡	c.1315+1G>A	p.R261P¶	c.782G>C	Yes	591	-4	No	
IVS12+1G>A‡	c.1315+1G>A	p.R261Q¶	c.782G>A	No	852	-1	No	
IVS12+1G>A‡	c.1315+1G>A	p.R261Q¶	c.782G>A	No	408	16	No	
IVS12+1G>A‡	c.1315+1G>A	p.R261Q¶	c.782G>A	Yes	709	22	No	
IVS12+1G>A‡	c.1315+1G>A	p.R261Q¶	c.782G>A	No	577	21	No	
IVS12+1G>A‡	c.1315+1G>A	p.Y277D¶	c.829T>G	No	557	-18	No	
IVS12+1G>A‡§	c.1315+1G>A	p.P281L‡	c.842C>T	Yes	950	22	No	
IVS12+1G>A‡§	c.1315+1G>A	p.P281L‡	c.842C>T	No	650	19	No	
IVS12+1G>A‡	c.1315+1G>A	p.P281L‡	c.842C>T	Yes	469	7	No	
IVS12+1G>A‡	c.1315+1G>A	p.A300S||	c.898G>T	Yes	321	62	Yes	positive
IVS12+1G>A‡	c.1315+1G>A	p.L348V||	c.1042C>G	Yes	593	33	Yes	positive
IVS12+1G>A‡	c.1315+1G>A	p.L367Pfs*27‡	c.1099_1100insC	Yes	397	-5	No	
IVS12+1G>A‡	c.1315+1G>A	p.L367Pfs*27‡	c.1099_1100insC	No	934	10	No	
IVS12+1G>A‡	c.1315+1G>A	p.A403V||	c.1208C>T	Yes	314	85	Yes	positive
IVS12+1G>A‡	c.1315+1G>A	p.R408W‡	c.1222C>T	No	890	-1	No	
IVS12+1G>A‡	c.1315+1G>A	p.Y414C||	c.1241A>G	No	896	63	Yes	positive
IVS12+1G>A‡	c.1315+1G>A	p.Y414C||	c.1241A>G	Yes	681	72	Yes	positive
IVS12+1G>A‡	c.1315+1G>A	p.Y414C||	c.1241A>G	Yes	576	83	Yes	positive
IVS12+1G>A‡§	c.1315+1G>A	p.Y414C||	c.1241A>G	No	535	56	Yes	positive
IVS12+1G>A‡§	c.1315+1G>A	p.Y414C||	c.1241A>G	No	390	45	Yes	positive

**Allele 1**		**Allele 2**		**Phe load†**	**T=0**	**Max Phe ↓ (%)**	**Extension phase**	
(Continued from previous page)								
IVS12+1G>A‡	c.1315+1G>A	p.Y414C‖	c.1241A>G	No	465	38	Yes	positive
p.L48S‖	c.143T>C	p.R158Q¶	c.473G>A	Yes	316	52	No	
p.L48S‖§	c.143T>C	p.R241C‖	c.721C>T	Yes	365	49	Yes	positive
p.L48S‖§	c.143T>C	p.R241C‖	c.721C>T	Yes	532	59	Yes	positive
p.L48S‖	c.143T>C	p.R261Q¶	c.782G>A	No	530	28	No	
p.L48S‖	c.143T>C	p.P281L‡	c.842C>T	Yes	525	63	Yes	positive
p.L48S‖	c.143T>C	p.R408W‡	c.1222C>T	No	815	36	Yes	positive
p.F55Lfs*6 ‡	c.165delT	p.P281L‡	c.842C>T	No	411	-7	No	
p.I65T‖	c.194T>C	p.L311P‡	c.932T>C	Yes	530	33	Yes	negative
p.I94del‡	c.283_285delATC	p.P314H‡‡	c.941C>A	Yes	436	65	Yes	positive
p.I94del‡	c.283_285delATC	p.Y414C‖	c.1241A>G	No	521	72	Yes	positive
p.A104D††	c.311C>A	p.A104D††	c.311C>A	Yes	660	57	Yes	positive
p.A104D††	c.311C>A	p.R158Q¶	c.473G>A	No	646	31	Yes	positive
p.A104D††	c.311C>A	p.R241C‖	c.721C>T	Yes	574	42	No	
p.A104D††	c.311C>A	p.R408W‡	c.1222C>T	Yes	486	34	Yes	positive
p.R111*‡	c.331C>T	p.R408W‡	c.1222C>T	No	766	14	No	
p.R158Q¶	c.473G>A	p.R158Q¶	c.473G>A	No	633	34	Yes	negative
p.R158Q¶	c.473G>A	p.R158Q¶	c.473G>A	No	535	5	No	
p.R158Q¶	c.473G>A	p.V190A‖	c.569T>C	No	327	53	Yes	positive
p.R158Q¶	c.473G>A	p.V190A‖	c.569T>C	Yes	511	52	Yes	positive
p.R158Q¶	c.473G>A	p.R243*‡	c.727C>T	No	323	4	No	
p.R158Q¶	c.473G>A	p.R261P¶	c.782G>C	No	1348	6	No	
p.R158Q¶	c.473G>A	p.Y277D¶	c.829T>G	Yes	257	40	Yes	negative
p.R158Q¶	c.473G>A	p.R408W‡	c.1222C>T	Yes	655	-1	No	
p.R158Q¶	c.473G>A	p.Y414C‖	c.1241A>G	No	366	68	Yes	positive
p.R158Q¶	c.473G>A	p.Y414C‖	c.1241A>G	Yes	362	74	Yes	positive
p.I174N¶§	c.521T>A	p.P211T††	c.631C>A	Yes	360	67	Yes	positive
p.I174T¶	c.521T>C	p.F299C‡	c.896T>G	No	1049	-24	No	
p.E178G‖	c.533A>G	p.Y343C¶	c.1028A>G	Yes	353	64	Yes	positive
p.W187*‡	c.561G>A	p.W187*‡	c.561G>A	No	505	17	No	
p.W187*‡	c.561G>A	p.T193I††	c.578C>T	No	365	62	Yes	positive
p.W187*‡	c.561G>A	p.L367Pfs*27‡	c.1099_1100insC	Yes	689	-2	No	
p.V190A‖	c.569T>C	p.S231F‡	c.692C>T	Yes	448	41	Yes	positive
p.V190A‖	c.569T>C	p.L311P‡	c.932T>C	Yes	592	76	No	
p.L197Lfs‡	c.590_611del	p.D415N‖	c.1243G>A	Yes	302	73	Yes	positive
p.C203Y¶	c.608G>A	p.L348V‖	c.1042C>G	Yes	486	25	No	
p.P211Hfs‡	c.632delC	p.S231P‡	c.691T>C	Yes	474	-8	No	
**p.T238A††**	c.712A>G	p.P281L‡	c.842C>T	Yes	549	59	Yes	positive
p.R243*‡	c.727C>T	p.R261Q¶	c.782G>A	No	620	39	Yes	negative
p.R243*‡§	c.727C>T	p.S349P‡	c.1045T>C	Yes	719	37	No	
p.R243*‡§	c.727C>T	p.S349P‡	c.1045T>C	Yes	693	22	No	
p.R243*‡§	c.727C>T	p.S349P‡	c.1045T>C	Yes	352	31	No	
p.R243*‡§	c.727C>T	p.A403V‖	c.1208C>T	Yes	515	87	Yes	positive
p.R243*‡§	c.727C>T	p.A403V‖	c.1208C>T	Yes	420	74	No	
p.R243X‡	c.727C>T	p.Y414C‖	c.1241A>G	Yes	634	49	Yes	positive
p.R243*‡	c.727C>T	**p.E440*‡**	c.1318G>T	No	560	21	No	
p.R252W‡	c.754C>T	p.P281L‡	c.842C>T	No	882	-2	No	
p.R261P¶	c.782G>C	p.Y414C‖	c.1241A>G	No	572	30	Yes	positive

(Continued from previous page)

Allele 1		Allele 2		Phe load†	T=0	Max Phe ↓ (%)	Extension phase	
p.R261Q¶§	c.782G>A	p.R261Q¶	c.782G>A	No	758	25	No	
p.R261Q¶§	c.782G>A	p.R261Q¶	c.782G>A	Yes	510	7	No	
p.R261Q¶	c.782G>A	p.E280K‡	c.838G>A	Yes	654	25	No	
p.R261Q¶	c.782G>A	p.E280K‡	c.838G>A	No	1476	25	No	
p.R261Q¶	c.782G>A	p.P281L‡	c.842C>T	No	1237	29	No	
p.R261Q¶	c.782G>A	p.P281L‡	c.842C>T	Yes	488	79	Yes	positive
p.G272*‡	c.814G>T	p.Y414C‖	c.1241A>G	Yes	462	84	Yes	positive
p.Y277D¶§	c.829T>G	p.P314H††	c.941C>A	Yes	206	33	Yes	positive
p.Y277D¶§	c.829T>G	p.P314H††	c.941C>A	Yes	213	68	No	
p.E280K‡	c.838G>A	p.Y414C‖	c.1241A>G	Yes	481	73	Yes	positive
p.P281L‡§	c.842C>T	p.P281L‡	c.842C>T	No	581	-6	No	
p.P281L‡§	c.842C>T	p.P281L‡	c.842C>T	No	1317	4	No	
p.P281L‡	c.842C>T	p.P281L‡	c.842C>T	No	1020	15	No	
p.P281L‡	c.842C>T	p.A300S‖	c.898G>T	Yes	480	66	Yes	positive
p.P281L‡	c.842C>T	p.Y356*‡	c.1068C>G	Yes	1181	38	Yes	negative
p.P281L‡§	c.842C>T	p.R413P‡	c.1238G>C	No	921	9	No	
p.P281L‡§	c.842C>T	p.R413P‡	c.1238G>C	No	640	-1	No	
p.A300S‖	c.898G>T	p.R408W‡	c.1222C>T	Yes	260	42	Yes	positive
p.G352Vfs*48 ‡	c.1055delG	p.G352Vfs*48 ‡	c.1055delG	No	570	60	Yes	negative
p.K363Nfs*37 ‡§	c.1089delG	p.K363Nfs*37 ‡	c.1089delG	No	817	3	No	
p.K363Nfs*37 ‡§	c.1089delG	p.K363Nfs*37 ‡	c.1089delG	Yes	576	19	No	
p.L367Pfs*27 ‡	c.1099_1100insC	p.Y414C‖	c.1241A>G	No	458	78	Yes	positive
p.R408Q††	c.1223G>A	p.R408Q††	c.1223G>A	Yes	315	36	Yes	positive
p.Y414C‖	c.1241A>G	p.Y414C‖	c.1241A>G	Yes	339	49	Yes	positive

† Phe supplementation, ‡Putative null mutation, § siblings/family, ¶ unclassified mutation, ‖ mutation associated with long-term BH4 responsiveness, †† mutation in this study newly associated with long-term BH4 responsiveness, novel mutations are in bold.
